# Effect of varying recovery intensities on power outputs during severe intensity intervals in trained cyclists during the Covid-19 pandemic

**DOI:** 10.1007/s11332-023-01050-2

**Published:** 2023-02-16

**Authors:** Alan Chorley, Kevin L. Lamb

**Affiliations:** Department of Sport and Exercise Sciences, University of Chester, Chester, CH1 4BJ UK

**Keywords:** Recovery-W′, Modelling, Fatigue, Self-selected, Cycling

## Abstract

**Purpose:**

The study aimed to investigate the effects of different recovery intensities on the power outputs of repeated severe intensity intervals and the implications for W′ reconstitution in trained cyclists.

**Methods:**

Eighteen trained cyclists (FTP 258.0 ± 42.7 W; weekly training 8.6 ± 1.7 h∙week^−1^) familiar with interval training, use of the Zwift® platform throughout the Covid-19 pandemic, and previously established FTP (95% of mean power output from a 20-min test), performed 5 × 3-min severe intensity efforts interspersed with 2-min recoveries. Recovery intensities were: 50 W (LOW), 50% of functional threshold power (MOD), and self-selected power output (SELF).

**Results:**

Whilst power outputs declined as the session progressed, mean power outputs during the severe intervals across the conditions were not different to each other (LOW 300.1 ± 48.1 W; MOD: 296.9 ± 50.4 W; SELF: 298.8 ± 53.3 W) despite the different recovery conditions. Mean power outputs of the self-selected recovery periods were 121.7 ± 26.2 W. However, intensity varied during the self-selected recovery periods, with values in the last 15 s being greater than the first 15 s (*p* < 0.001) and decreasing throughout the session (128.7 ± 25.4 W to 113.9 ± 29.3 W).

**Conclusion:**

Reducing recovery intensities below 50% of FTP failed to enhance subsequent severe intensity intervals, suggesting that a lower limit for optimal W′ reconstitution had been reached. As self-selected recoveries were seen to adapt to maintain the severe intensity power output as the session progressed, adopting such a strategy might be preferential for interval training sessions.

## Introduction

The outcome of mass start cycle races is often determined by a competitor breaking free of their rivals in a decisive move. However, this pivotal event is usually preceded by a series of short (5–15 s) sprint efforts and immediately followed by longer (30–300 s) high intensity efforts each separated by periods of recovery [1]. Indeed this ability to recover and repeat high intensity efforts is a discriminating factor separating the best riders from the rest [2]. Such efforts are described as being within the ‘severe’ intensity domain [3] and above the individual’s critical power (CP), which reflects the highest power output that can be sustained by wholly aerobic means [4] and marks the boundary between the severe and heavy intensity domains. The amount of effort (work) which the cyclist can perform above CP (W′) is finite [5] and will limit the duration and power output which can be sustained in severe high intensity efforts.

The underlying physiology of W′ is only partly understood and is believed to be dependent upon intra-muscular phosphocreatine (PCr) and glycogen stores [6] and an accumulation of fatiguing metabolites [7, 8]. Exercise in the severe domain depletes W′ proportionally to the power output above CP [9] and independently of the rate of utilisation [10]. However, the kinetics of W′ reconstitution are more complex, occurring when power output falls below CP [11], and demonstrating a curvilinear profile with respect to both time [12] and duration [13]. Skiba et al. [14] proposed a mono-exponential model of W′ reconstitution (W′_bal_ model) predicting the balance of W′ at a point in time (Eq. 1).

1 $$W^{\prime}_{bal} = W^{\prime} - \int\limits_{0}^{t} {\left( {W^{\prime}_{\exp } } \right)} \cdot \left( {e^{{{{ - \left( {t - u} \right)} \mathord{\left/ {\vphantom {{ - \left( {t - u} \right)} {\tau_{{w^{\prime}}} }}} \right. \kern-0pt} {\tau_{{w^{\prime}}} }}}} } \right) \cdot du$$

2 $$\tau W^{\prime} = 546 \cdot e^{\left( { - 0.01 \cdot D_{CP} } \right)} + 316$$

where W′_bal_ = balance of W′ at time *t* (J); W′ = initial known W′ (J); *W′*_exp_ = total W′ expended (*J*); t–u = recovery duration (s); *τ*_*W*′_ = W′ reconstitution time constant (s); D_CP_ = difference between the known CP and recovery power (W).

Severe intensity interval training sessions individualised to account for differing rates of W′ reconstitution using the W′_bal_ model have been shown to increase CP in trained athletes [15]. The rate of W′ reconstitution is dependent upon the time constant (*τ*) which can be estimated using Eq. 2 [14], indicating that *τ* (and thus, the rate of W′ reconstitution) is dependent on the difference between CP and recovery power output (D_CP_) [14, 16, 17]. This theoretical equation also suggests that the W′ reconstitution rate no longer improves once recovery power output is lower than 316 W below CP; however, this has yet to be verified experimentally. It has been suggested that the W′_bal_ model underestimates the rate of W′ reconstitution in both elite [16] and non-elite cyclists [18, 19]. Furthermore, recent investigations have suggested a bi-exponential model better represents W′ reconstitution kinetics [17, 20], although such a model accounting for varying power output has not been proposed. The reconstitution kinetics of W′ appear to be unaffected by sex [17, 21, 22], body mass, or age per se [23] but is relative to measures of aerobic fitness such as CP and maximum oxygen uptake (V̇O_2max_) [20, 23]. Published studies investigating W′ reconstitution [14, 18, 19, 20, 22] invariably have a fixed power output (intensity) for recovery phases. None have considered the merit of applying a self-selected intensity, which, based on anecdotal evidence (whereby we have observed cyclists intuitively vary their recovery efforts when left to their own volition), may enhance subsequent power output. However, recovery between intermittent efforts is a complex interaction of physiological processes such as PCr restoration and H^+^ removal, which may be optimal at low intensities [24], and priming for the subsequent effort which may benefit from a higher intensity recovery due to an elevated oxygen uptake [20]. Thus, a variable, or increasing, or self-selected recovery intensity could offer both an initial increase in PCr restoration and a priming effect, together providing an improvement in subsequent severe intensity performance.

Changes in power output demands are met by changes in cardiac output to facilitate oxygen delivery to the working muscle. During sub-maximal exercise, a linear response is observed between heart rate (HR) and power output [25]; however, above CP neither HR nor V̇O_2_ stabilise and instead continue to rise until the limit of tolerance [26]. Both HR and V̇O_2_ exhibit a delayed response to increased power output demands and the kinetics of both are strongly correlated with each other when cycling [27]. Furthermore, faster V̇O_2_ and HR kinetics in response to an increase in demand are associated with improved performance in exercise above CP [28]. Similarly, during recovery, the difference between HR at the end of a bout of severe exercise and after 2 min of recovery has been shown to correlate to the amount of W′ reconstituted during that period [23]. Indeed, V̇O_2_ during recovery can be considered a proxy measurement for PCr restoration.

Whilst together CP and W′ can predict and describe physiological performance and CP in particular is arguably the most informative physiological threshold reported in the scientific literature [29], many practitioners and coaches have yet to embrace it, instead preferring the use of the single pseudo-physiological measurement ‘functional threshold power’ (FTP) owing to the simplicity of its measurement (not requiring laboratory testing) [30, 31]. Unsurprisingly, therefore, on-line home-based cycle training applications, such as Zwift® (Zwift Inc., Long Beach, CA, US), which have increased in popularity due in part to the Covid-19 pandemic [32], have similarly used FTP rather than physiological measurements such as CP. Moreover, whilst the Zwift® application provides a gameplay environment based on its physics engine, it can also be used as a convenient method of applying test protocols to ‘smart trainers’ and recording their output. Several direct drive smart trainers\cycles of this type have previously been used in laboratory-based studies and reported as valid and reliable [33, 34, 35].

FTP, defined as the maximum power output that can be sustained for around 1 h [36], was proposed as a proxy measure of laboratory-derived measures such as CP and maximal lactate steady state that aim to identify the demarcation between the heavy and severe intensity domains [3]. FTP is usually estimated by multiplying the mean power output of a 20-min maximum effort test by 0.95 [31] and has been shown to be strongly correlated to CP [37, 38] and whilst a recent study has reported a small bias of 7 W (CP > FTP) [37], others have reported no significant difference at a group level [38].

The primary aim of this study was, therefore, to investigate the effects of different recovery intensities below the FTP, including a self-selected recovery, on the power outputs and work done during repeated severe intensity efforts, and thus, the implications on W′ reconstitution, using readily available home-based cycle equipment and the Zwift® platform. Specifically, we hypothesised that self-selected recovery intensities would result in the greatest power output and work done through the session and that lower recovery intensities would provide no further benefit.

## Methods

### Participants

Following institutional ethical approval and in accordance with the declaration of Helsinki, 18 adult cyclists (male: *n* = 16; age 48.7 ± 10.8 years; stature 177.4 ± 4.5 cm; body mass 72.7 ± 6.6 kg; FTP 264.9 ± 39.1 W; female *n* = 2; age 49.5 ± 3.5 years; stature 168.9 ± 9.2 cm; body mass 63.0 ± 6.4 kg; FTP 203.0 ± 42.4 W) volunteered to participate in the study during the Covid-19 pandemic and provided written informed consent. Participants were all amateur cyclists training 8.6 ± 1.7 h∙week^−1^ and familiar with indoor training on the Zwift® platform and with high intensity interval sessions.

### Procedures

The study design followed a repeated measures randomised experimental approach, whereby participants completed a minimum of four testing sessions comprising a familiarisation session which was repeated as required, and three experimental sessions with different recovery power outputs. Experimental sessions were conducted in a randomised order. The final familiarisation session and the experimental sessions were completed within a 16-day period, with at least 2 days between sessions. Participants were requested to perform the Zwift® FTP test to determine their FTP if they had not done so in the 4 weeks prior to starting the experimental sessions. The Zwift® FTP test included an easy warm-up followed by a 5-min maximum effort and a further 10 min of easy pedalling before a 20-min maximum effort, whereby FTP was determined by multiplying the average power output of the maximum effort by 0.95 [39]. Participants were requested to perform each session at similar times of day, having avoided strenuous exercise for 24 h, caffeine for 4 h, and were 3 h postprandial. All sessions were undertaken at the participants’ homes on their own cycles and smart trainers, or smart ‘bikes’ which were certified by the manufacturers as reporting power output to an accuracy of within ± 2% (or better). The smart trainers and smart bikes were compliant with the regulated equipment requirements of the world governing body for cycle sport (UCI) and Zwift® for the Esports World Championships qualifying events [40, 41]. Zwift® session files were sent to each participant based on their pre-determined FTP. Sessions were performed using the Zwift® application which recorded power output, cadence and per second HR via an Ant + or Bluetooth chest strap in a.fit format data file. The files were then analysed for compliance to the instructed protocol. If power output during the high-intensity phases dropped below the participant’s individual FTP for more than 3 s, the participant was asked to repeat the session due to the partial recovery of W′ that could be facilitated within the heavy intensity domain.

### Session protocol

**Fig. 1:**
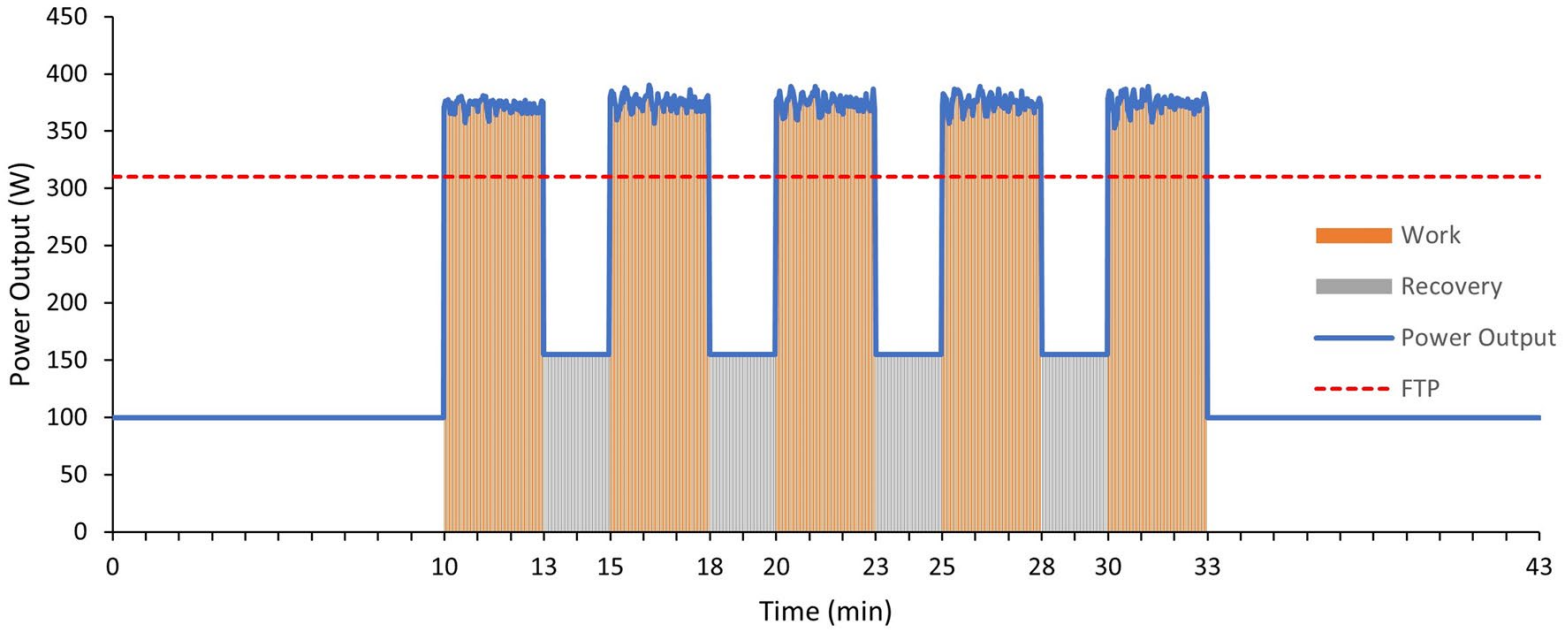
Example power profile of an experimental session with recovery at 50% of FTP (MOD) for a participant with a 310 W FTP. 2-min recovery periods were varied between sessions comprising LOW (50 W), MOD (50% of FTP) and SELF (self-selected variable power) intensities

Prior to each session, participants were asked to calibrate their smart trainer in accordance with the manufacturer’s instructions. Each session followed the same format (see Fig. 1) comprising a 10-min warm-up at 100 W followed by 5 × 3-min severe intensity interval efforts separated by 2-min recoveries, ending with a further 10-min cool down at 100 W. Participants were instructed to perform the 3-min efforts to produce the highest average power output during each individual effort and to sustain the highest average power output possible across the five efforts, with instant or 3-s averaged power visible on-screen. As the independent variable, the intensity of the 2-min recovery was varied in a random order between trials. That is, a fixed power output of 50 W (LOW), a fixed power output of 50% of the individual’s FTP (MOD) and a self-selected power output accommodating passive recovery if preferred (SELF). Session files were constructed such that all steady state phases (warm-up, LOW and MOD recoveries, and cool down) within sessions were performed using the ‘erg mode’ whereby the resistance of the trainer was adjusted automatically to elicit the desire power output, and non-steady state (3-min severe efforts and SELF recoveries) was performed at a fixed resistance allowing power output to vary.

### Statistical analysis

Descriptive statistics (mean ± SD) were calculated for all dependent variables and the normality of their distributions was checked using the Shapiro–Wilk test. Two-way repeated measures ANOVA (recovery condition x interval number) was used to assess the differences in power output, work done and end HR (i.e. HR at the end of each interval effort) due to the independent variables. Similarly, two-way repeated measures ANOVA (recovery condition x recovery number) was used to assess differences in power output and minimum HR during recovery periods. A one-way repeated measures ANOVA was used to assess differences in the amount of work performed during the first interval effort to verify that participants did not pre-empt the different recovery conditions by altering their power output. Differences in absolute maximum HR between conditions were assessed with a one-way repeated measures ANOVA. Self-selected recovery characteristics were further investigated with a one-way repeated measures ANOVA to assess the effects of recovery number on mean power output during the recovery periods, and two-way repeated measures ANOVA (recovery condition x recovery number) were used to assess differences in power output between the first 15 s and the final 15 s of the self-selected recoveries. Sphericity was checked with Mauchly’s test and accounted for where necessary using the Greenhouse–Geisser adjustment. Significant effects were investigated with Bonferroni adjusted post hoc pairwise analysis where appropriate. Statistical significance was set at *P* < 0.05 throughout. All statistical analyses were performed using SPSS v.27 (IBM Corp., Armonk, NY, US).

## Results

### Recovery periods

The mean SELF intensity (121.7 ± 26.2 W) was greater (*p* < 0.001) than the LOW condition (57.2 ± 6.8 W) but not different to the MOD (127.2 ± 20.6 W; *p* = 0.28). There were significant main effects (*p* < 0.001) of recovery number and condition on minimum HR at the end of the recovery periods, with HR rising as the session progressed and differing across the three conditions, but no interaction effect (*p* = 0.72). Post hoc comparisons revealed minimum HR to be lower during the LOW condition than both MOD (mean difference: 13.6 bpm; *p* < 0.001) and SELF (mean difference: 11.2 bpm; *p* < 0.001), but not different between SELF and MOD conditions (mean difference: 2.4 bpm; *p* = 0.13) (see Table 1).

**Table 1:** Power outputs (mean ± SD) and minimum heart rates during recovery periods encountered during each recovery period for the LOW, MOD and SELF conditions

	Recovery number				
	1	2	3	4	Mean
MOD recovery power output (W)	127.9 ± 20.7	126.0 ± 20.3	127.8 ± 20.9	127.1 ± 21.5	127.2 ± 20.6
LOW recovery power output (W)	57.4 ± 6.8	57.9 ± 7.3	56.7 ± 7.2	56.8 ± 6.8	57.2 ± 6.8
SELF recovery power output (W)	128.7 ± 25.4	126.2 ± 25.5	118.05 ± 28.9	113.9 ± 29.2	121.7 ± 26.2
MOD min heart rate (bpm)	119.8 ± 13.2	124.3 ± 12.6	128.2 ± 14.1	130.3 ± 15.5	125.6 ± 14.1
LOW min heart rate (bpm)	105.8 ± 16.1	110.5 ± 15.4	114.8 ± 16.4	117.4 ± 17.1	112.1 ± 16.4
SELF min heart rate (bpm)	116.9 ± 12.2	122.7 ± 13.4	126.1 ± 13.8	127.6 ± 15.3	123.3 ± 14.0

### Self-selected recoveries

**Fig. 2:**
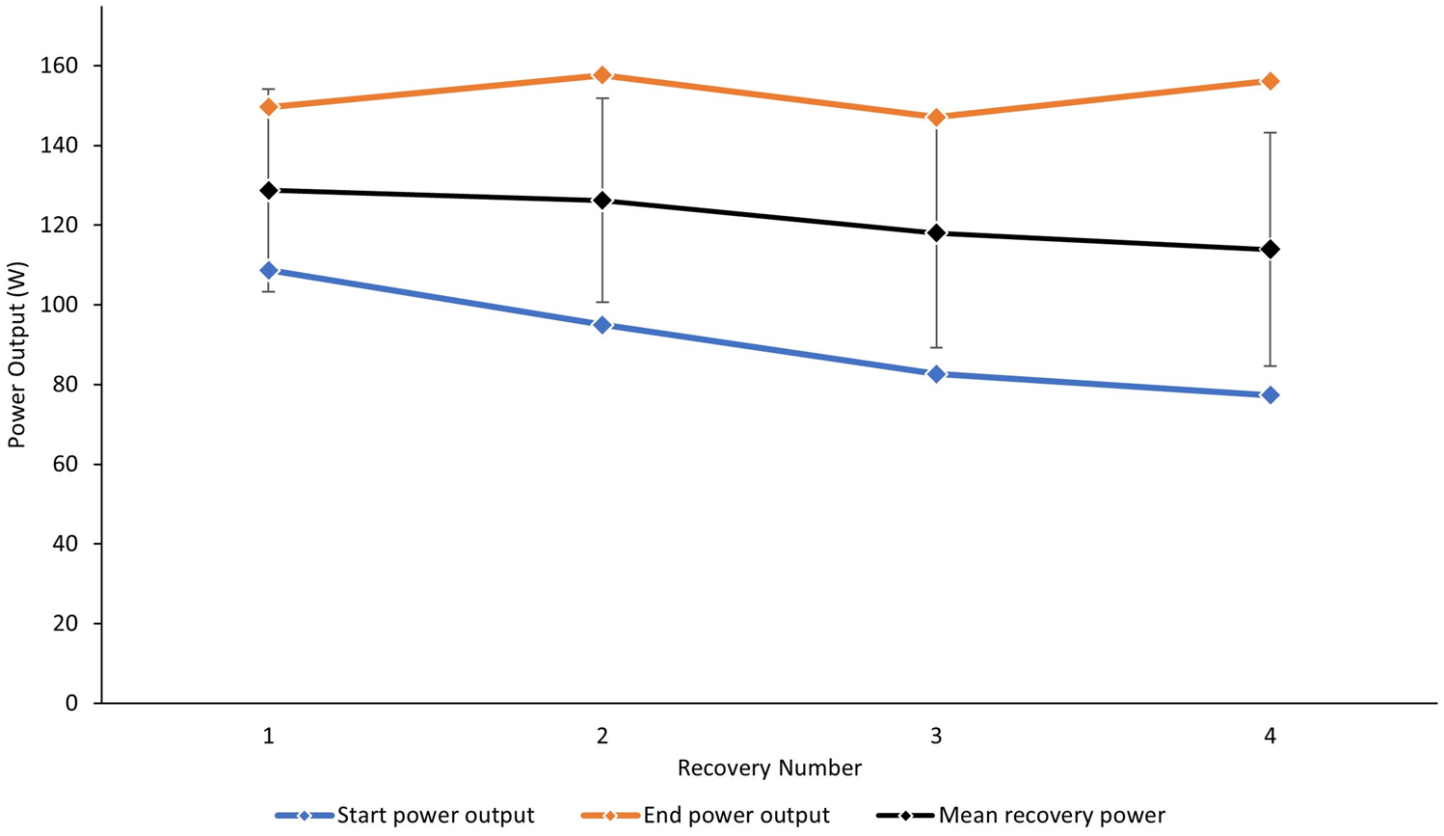
Self-selected recovery power outputs during the first 15 s (‘Start’) and the final 15 s (‘End’) of each recovery period. Black markers indicate the mean power output for the entirety of each recovery. Error bars removed from ‘start’ and ‘end’ power outputs for clarity

The mean power outputs generated during the self-selected recoveries varied due to the recovery number (*p* < 0.001), falling from 128.7 ± 25.4 W to 113.9 ± 29.3 W as the sessions progressed (see Fig. 2). The effect of intra-recovery phase (first 15 s of recovery against last 15 s of recovery) on power output was significant (*p* < 0.001), with the values in the last 15 s being greater than the initial 15 s of every recovery period. There was also a significant main effect of interval number with power output falling as the session progressed (*p* < 0.001), and an interaction effect (*p* < 0.001), reflecting an increasing difference between power outputs at the start and end of each recovery phase as the session progressed (Fig. 2).

### Severe efforts

**Fig. 3:**
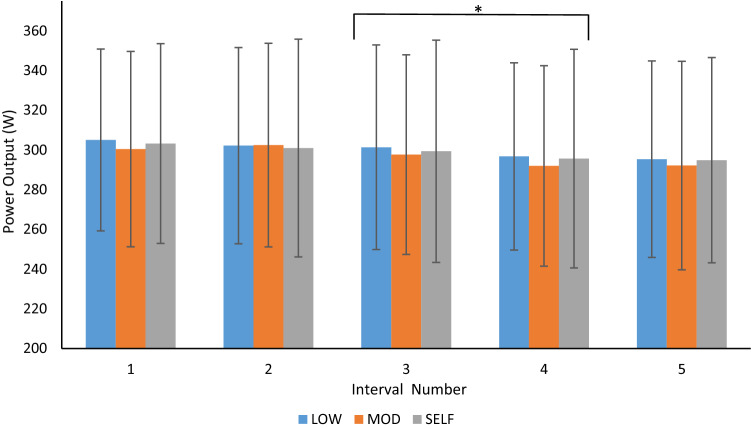
Mean power (± SD) output of 3-min severe intensity interval efforts in LOW, MOD and SELF conditions. * *p* < 0.001 difference between adjacent intervals

Mean power outputs during severe interval efforts (see Fig. 3) were 300.1 ± 48.1 W (LOW), 296.9 ± 50.4 W (MOD) and 298.8 ± 53.3 W (SELF), all of which were significantly greater than FTP: 258.0 ± 42.7 W (*p* < 0.001). Neither the effect of condition (*p* = 0.15) nor the condition x interval number interaction effect (*p* = 0.52) on power output was significant, but there was a main effect of interval number (*p* < 0.001), with power outputs reducing as the session progressed across the five severe intervals. Post hoc analysis revealed a significant decline in power output (*p* < 0.001) between intervals three and four of 4.6 ± 6.5 W. The total work completed during the first severe interval prior to the start of the recovery periods was not different between conditions (LOW: 54.9 ± 8.2 kJ; MOD: 54.1 ± 8.9 kJ; SELF: 54.6 ± 9.1 kJ;* p* = 0.21). Similarly, total work done during the final four severe intervals (following the onset of the recovery periods) did not differ between conditions (LOW: 215.2 ± 35.5 kJ; MOD: 213.2 ± 36.6 kJ; SELF: 214.3 ± 38.8 kJ; *p* = 0.25).

The maximum HR recorded across sessions (169.6 ± 12.1 bpm, 169.4 ± 11.7 bpm and 168.9 ± 12.6 bpm for the LOW, MOD and SELF conditions, respectively) were not significantly different (*p* = 0.82). End HR (that recorded at the end of each severe interval) was found to vary across the five efforts (*p* < 0.001), rising progressively throughout the session, but did not vary as an effect of the recovery condition (*p* = 0.42) or the effort x condition interaction (*p* = 0.80).

## Discussion

The principal finding of this study was that when compared to recoveries performed at 50% of FTP (MOD), a lower recovery intensity failed to enhance the power outputs or work done in subsequent severe intensity efforts. Furthermore, freely chosen (and changeable) recovery intensities yielded similar mean power outputs to 50% of FTP, and similar HR responses in the subsequent severe intensity efforts. The maximum HR during the severe intensity efforts that were not different between recovery conditions indicates that high motivation was maintained throughout the trials [42], corroborating the lack of influence of the recovery intensity on subsequent performance due to the similar HR kinetic responses, which are thought dependent upon blood lactate levels, intensity and duration of efforts [26]. These findings suggest there is likely a lower boundary of recovery intensity beyond which there is no further benefit to subsequent severe intensity efforts. Previous regression modelling has suggested an asymptote beyond which the rate of W′ reconstitution can no longer be accelerated [22]; however, the absolute value of 316 W below CP proposed by Skiba et al. [14] would be considerably beyond the lower limit of 50% of FTP suggested by the current study.

Whilst FTP is within the heavy intensity domain [43], it does not equate to CP or any other physiological landmark [43], and thus cannot be used to determine values of CP and W′. It does, however, correlate strongly with CP [37, 44]. Moreover, in a cohort with similar FTP to the current study (249 ± 13 W versus 258 ± 43 W), Karsten et al. [37] reported a mean bias of 7 ± 13 W (CP being greater) and limits of agreement of − 19 to 33 W. As the high intensity efforts in the current study were performed considerably above the reported bias between CP and FTP (approximately 40 W above FTP), it can be assumed that they were exclusively within the severe intensity domain, and thus limited by the capacity of W′ and its reconstitution during the recovery phases. Work within the severe domain is characterised by increasing blood lactate and V̇O_2_ [3], neither of which can attain a steady state and represent a progressive loss of muscle efficiency [45], depletion of muscle PCr stores [11] and an accumulation of fatiguing metabolites such as inorganic phosphate and hydrogen ions [11]. Whilst all three recovery power outputs in the current study were performed at intensities much less than 76% of CP and, thus, well within the moderate intensity domain [46], it has been previously shown that recovery work rates just below the first lactate threshold do not provide optimal recovery for subsequent severe intensity efforts [12], suggesting that the first lactate threshold itself is not the critical point beyond which recovery does not improve. Hence, the lower boundary for the fastest recovery of W′ is likely between 50% of FTP and the first lactate threshold.

That the mean power output of recoveries at 50% of FTP (MOD) and self-selected intensities were not different explains the similar power outputs generated during the subsequent severe intervals; however, the manner of the self-selected recoveries was markedly different to that of the constant MOD recovery condition; power output was initially reduced before increasing as the recovery phase progressed. This pattern is consistent with recent suggestions of the occurrence of a bi-exponential recovery of W′ [17, 20] in which a fast component is highly dependent upon the first 30 s of recovery [17]. This fast recovery phase shares a similar time course to that of PCr restoration [47] and is likely facilitated by the low power outputs at the outset of the MOD recoveries, given that PCr restoration has been shown to be greatest during passive recovery [48]. It has been suggested that the slow recovery phase of the bi-exponential model is linked to the removal of the accumulated metabolites such as lactate, inorganic phosphates and hydrogen ions [17]. Interestingly, no participants in the present study chose passive rest during any point of their self-selected recoveries. The increasing power output generated towards the end of the recoveries may be an instinctive or learned preparatory action to elevate oxygen uptake prior to the start of the subsequent high intensity efforts. Such an elevated V̇O_2_ at the outset of severe intensity work has been found to reduce oxygen deficit [20] and enable an increased W′ expenditure. Moreover, the reduction in overall self-selected recovery intensities as the session progressed (from 129 ± 25 W to 114 ± 29 W) suggests a further natural response to the progressive depletion of W′ and the slowing of W′ reconstitution which occurs with successive bouts [19]. The large between-subject differences observed have been previously reported in the recovery kinetics following severe exercise [14, 18, 23], and it is likely that such individual variability influences the self-selected intensities and explain the large variations (SD) seen in the current study. As such, self-selected recoveries may be preferential to fixed intensities prescribed for training sessions where sustaining maximum power outputs over repeated efforts is the session goal.

## Limitations and future research

The current study was performed during the Covid-19 pandemic with participants performing sessions at home using their own equipment. Participants were familiar with the FTP concept and undertaking FTP tests under such conditions, however, whilst the study has linked the findings to the CP model and W′ reconstitution, direct calculations of W′ expenditure cannot be made without first determining CP and W′. Such tests may be better performed under laboratory conditions owing to their demanding nature and the validity requirements needed to quantify W′ and its reconstitution for the purposes of refining W′ balance models. As such, future laboratory-based research is needed to quantify and apply the current findings to an improved model of W′ reconstitution, whilst home- or field-based studies using similar equipment to the present study can readily validate concepts and models of W′ reconstitution.

## Conclusion

The current study demonstrates that the performance of repeated efforts is not improved by reducing power outputs below approximately 50% of FTP, which is important for the purpose of modelling W′ reconstitution. Self-selected recoveries within the moderate intensity domain demonstrated a variable but consistent pattern which is likely a natural and effective response to the progressive depletion of W′ following repeated severe intensity efforts, potentially leading to optimal reconstitution of W′ when the recovery duration is known. Repeated severe intensity efforts can be the decisive factor in many forms of cycle racing, and current on-board cycle computers can relay the estimation of W′ balance following such efforts to a cyclist in real time, thus influencing race tactics and outcomes. Consequently, the incorporation of a lower limit of recovery power output could improve W′ reconstitution models, likewise enhancing the tactical feedback provided to cyclists.

## Data Availability

Data are available upon request from the corresponding author.
